# Pigmentation of the Cornea Secondary to Tinted Soft Contact Lens Wear

**DOI:** 10.1155/2012/852304

**Published:** 2012-01-30

**Authors:** Natasha Spiteri, Anshoo Choudhary, Stephen Kaye

**Affiliations:** St. Paul's Eye Unit, Royal Liverpool University Hospital, Prescot Road, Liverpool, L7 8XP, UK

## Abstract

*Purpose*. To report a case of pigmented corneal iron lines following use of tinted soft contact lenses (CL). *Methods*. A retrospective case report. *Results*. A 16-year-old girl was referred with suspected CL-related keratopathy OU, having recently switched to tinted soft monthly disposable CLs (8.4/14.0 −3.00 OD, −3.25 OS Aquamarine SofLens Natural Colours, Bausch and Lomb, New York, USA). Both corneas exhibited symmetric superficial corneal pigmented iron lines, which gradually disappeared following discontinuation of CL wear. *Conclusions*. Pigmented corneal rings have been reported in normal ageing corneas, in certain pathological conditions, and in association with altered corneal topography following LASIK and orthokeratology. We suspect a poorly fitting CL resulted in localised tear pooling between the CL and cornea, and subsequent iron pigment deposition, similar to that seen with orthokeratology. Cosmetic CLs bought via the Internet can be used in an unsupervised manner, with possible impacts on visual function and potential complications.

## 1. Introduction

 We report the case of a young female with apparent bilateral, symmetric pigmented iron lines on the cornea following the use of daily wear tinted soft monthly disposable CLs. Corneal iron lines have been reported in association with altered corneal topography and tear pooling in orthokeratology; however, to our knowledge, they have not been reported in association with tinted soft monthly CLs to date.

## 2. Methods

A retrospective case report format is used.

## 3. Results

A 16-year-old girl was referred with a history of a suspected CL-related keratopathy in both eyes, shortly after having switched to tinted soft monthly disposable CLs (8.4/14.0 −3.00 OD, −3.25 OS Aquamarine SofLens Natural Colours, Bausch and Lomb, New York).

**Figure 1:**
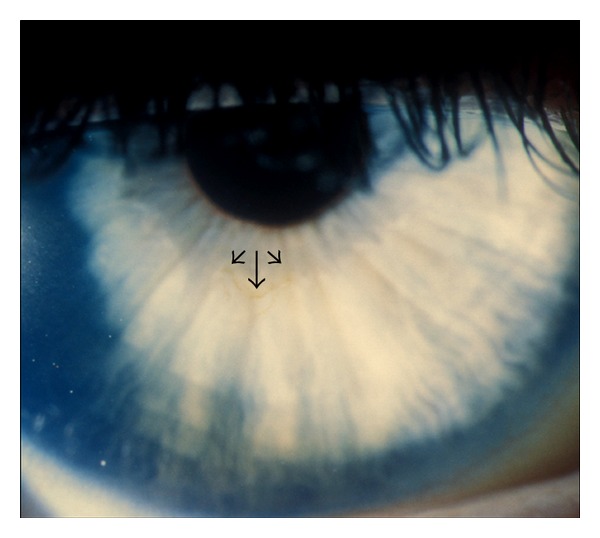
Line of corneal pigmentation (arrowheads).

Best corrected visual acuities (BCVA) were 6/9 OD and 6/5 OS. Both corneas exhibited symmetric superficial corneal pigmented iron lines (Figure 1). No other ocular surface abnormality was present. Following discontinuation of CL wear the pigmented lines disappeared over the subsequent three months and BCVA improved to 6/4 OU. 

## 4. Discussion

 Pigmented corneal rings have been reported in normal ageing corneas Hudson-Stahli lines and also in certain pathological conditions such as the Fleischer ring in keratoconus [1]. Corneal iron lines have also been associated with altered corneal topography and tear pooling following procedures such as LASIK [2] and orthokeratology [3].

Tinted CLs have been reported to induce several morphological and physiological changes in the cornea as well as having a negative impact on visual function. Previously noted morphological changes include a distinct ring-shaped pattern of irregular astigmatism with concentric areas of relative steepening and flattening, coined “the annular tinted contact lens syndrome.” These changes were rapidly reversible on discontinuing tinted lens use [4].

We suspect that in our case, a poorly fitting CL resulted in a localised pooling of tears between the CL and the corneal surface and subsequent deposition of iron pigment lines similar to that seen with orthokeratology.

In this case, the CLs were purchased over the Internet, and were being used in an unsupervised manner. This may often be the case, especially where users are buying plano-tinted CLs purely for cosmetic use via the Internet, and not from a CL specialist. This may present increasing problems to ophthalmic practitioners, in view of the possible impacts on visual function and potential complications associated with these lenses.
